# Delayed distal pancreatectomy for isolated complete pancreatic disruption secondary to “trivial” blunt abdominal injury: A case report and literature review

**DOI:** 10.1002/ccr3.6295

**Published:** 2022-09-06

**Authors:** Girmaye Tamrat, Segni Kejela

**Affiliations:** ^1^ Department of Surgery College of Health Sciences Addis Ababa University Addis Ababa Ethiopia

**Keywords:** blunt abdominal injury, distal pancreatectomy, isolated pancreatic injuries

## Abstract

Pancreatic injury is a formidable diagnostic and therapeutic challenge owing to its relative rarity. Most injuries are from motor vehicle related injuries in blunt trauma patients. We present a 22‐year‐old male patient presented after sustaining a kick to the abdomen. He developed progressive abdominal pain with vomiting with delayed generalization of the pain and involuntary guarding. On initial exploratory laparotomy, suction drainage was inserted, and patient underwent delayed spleen sparing distal pancreatectomy on the 25th post‐admission day. Patient had smooth postoperative course and was discharged on the 7th postoperative day.

## Graphical abstract

Of the abdominal viscera injuries, pancreatic trauma cases are one of the most difficult to diagnose owing to the delayed and unspecific clinical presentations, and lower sensitivity of contrast enhanced CT scan. A high index of suspicion is warranted especially in patients with progressively worsening clinical conditions.

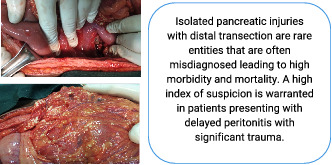

## 1 INTRODUCTION

Since the very first report of pancreatic trauma in 1827 by Travers, the reported incidences of pancreatic trauma have remained very low.^1^, ^2^, ^3^ Pancreatic injury reports have been seen in slightly higher proportion in blunt than penetrating abdominal trauma cases.^4^, ^5^ Difficulty in diagnosis and management of these cases has always been a formidable challenge, especially in patients with isolated pancreatic injuries.^6^ Here, we present a challenging case of a young patients presenting after blunt abdominal injury with severe pancreatic trauma with review of literature on the challenges in the management of such cases.

## 2 CASE PRESENTATION

A 22‐year‐old male patient presented to the emergency department of the Princesss Zewditu Memorial Hospital 9 h after sustaining a kick to the abdomen from unknown assailant. He had epigastric abdominal pain with three episodes of vomiting of ingested matter. There was no history of alcohol ingestion at presentation and reported no history of chronic medical illness.

On initial physical examination, pulse rate was 80 beats/minutes with blood pressure of 130/80 mmHg, and the respiratory rate was 22 breath/minute. Abdominal examination showed, direct epigastric tenderness with no guarding or rigidity. Other body systems examinations were non‐reviling.

Initial investigations showed, a White blood count (WBC) of 9800/mm^3^ with Neutrophil of 79%, Hemoglobin 14.9 g/dl, Platelet of 319,000/mm^3^. Erect chest X‐ray and Abdominal Ultrasound were normal.

The patient was assessed as blunt abdominal trauma with suspected solid organ injury and was admitted to the general ward for follow‐up with vital sign monitoring, analgesia, and a serial hematocrit. On the second day of admission, abdominal pain worsened and generalized to the whole abdomen with associated a complaint of fever and abdominal distention. On physical examination, pulse rate was 96 bpm, blood pressure 120/70 mmHg, respiratory rate 24 breaths/minute, and the axillary temperature was 37.7°C. Abdomen had involuntary guarding with direct and rebound tenderness with hypo‐active bowel sounds. On investigations, WBC was 15,200/mm^3^ with Neutrophil of 85%, Hgb 14.7 g/dl, Platelet 249,000/mm^3^. After the evaluation was completed, the patient was taken to the operation theater for exploratory laparotomy.

**FIGURE 1:**
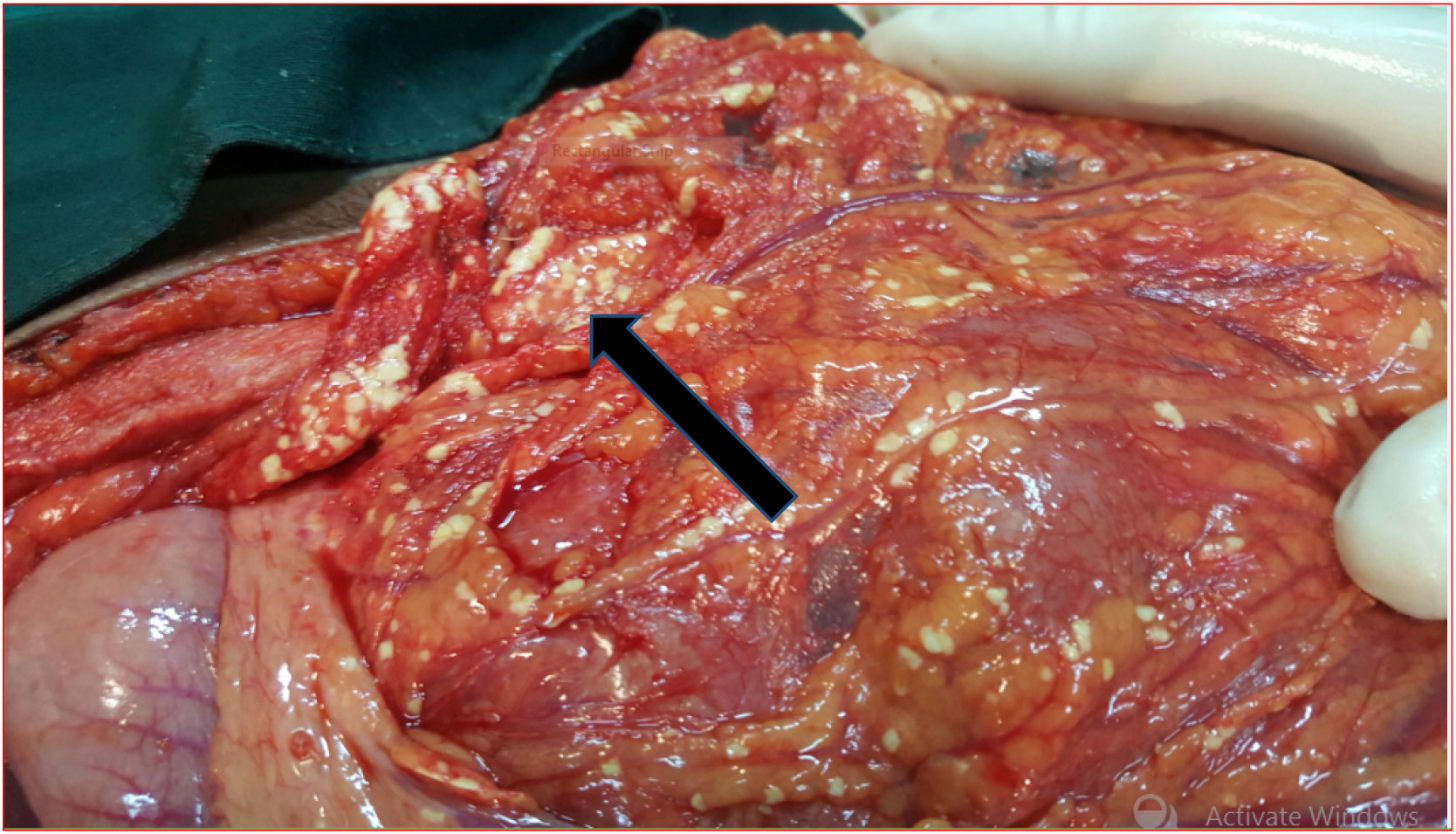
Diffuse Omental fat suponification

**FIGURE 2:**
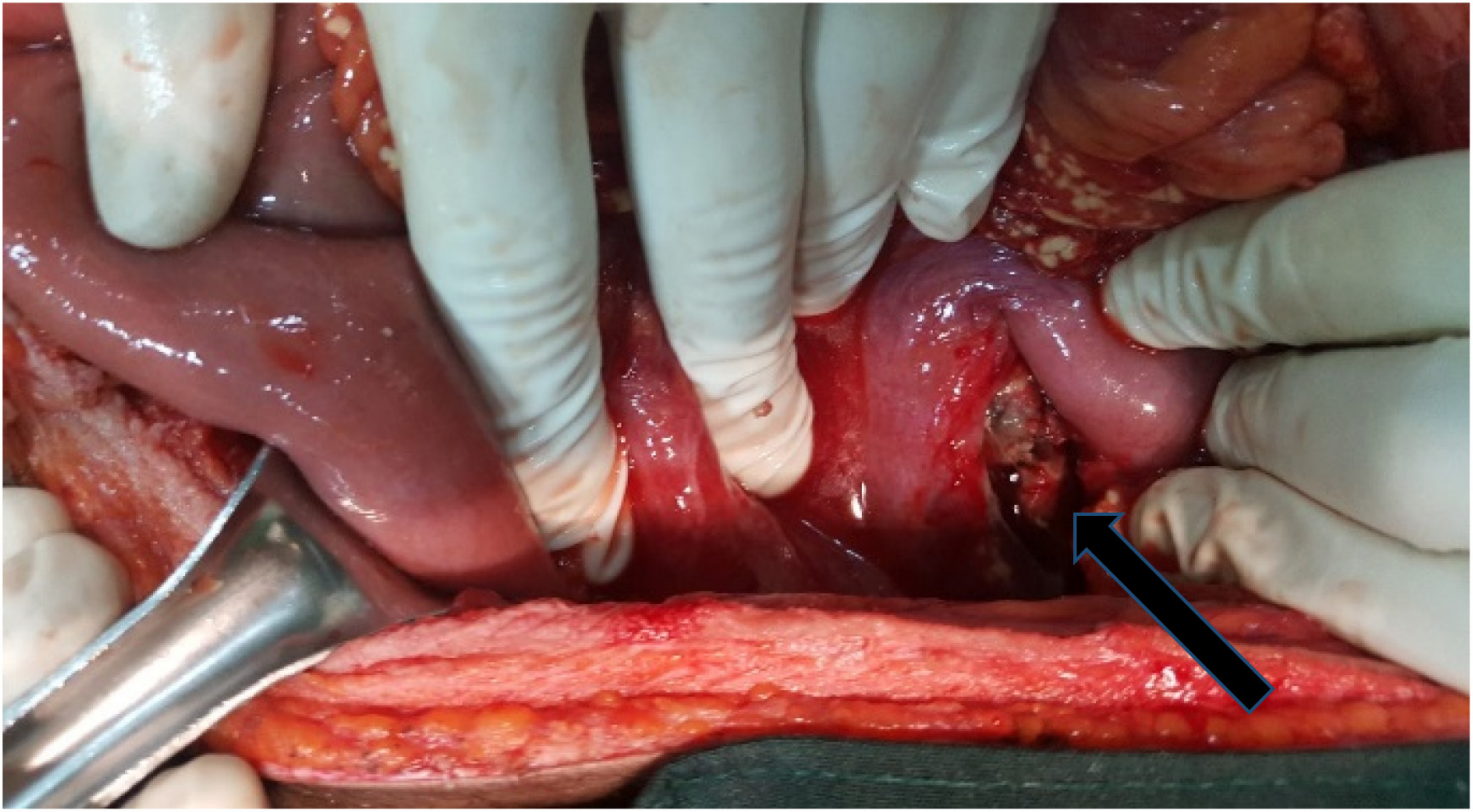
Mesentric fat suponification with pancreatic body necrotic transection

During the exploratory laparotomy, there was about 1800 ml hemorrhagic fluid in the general peritoneum. There were multiple sites of saponification on the greater omentum, small and large bowel mesentery fat (Figure 1). The lesser sack was opened, and pancreas was inflamed, with area of necrosis (darkened and almost eaten up) at junction of body and neck area (Figure 2). With intraoperative diagnosis of pancreatic injury, the hemorrhagic fluid was sacked out and large bore suction drainage tube left in the lesser sac and abdomen closed. Patient was transferred to the general surgical ward.

Postoperative investigations showed, amylase of 769 U/L (8.9* elevated) and lipase of 297 U/L (5.7* elevated), ionized calcium of 0.92 mmol/L (low) with all other laboratory parameters in normal range. The drainage output maintained at around 1200 ml of clear pancreatic fluid daily with stable vital signs and resolved signs of generalized peritonitis.

**FIGURE 3:**
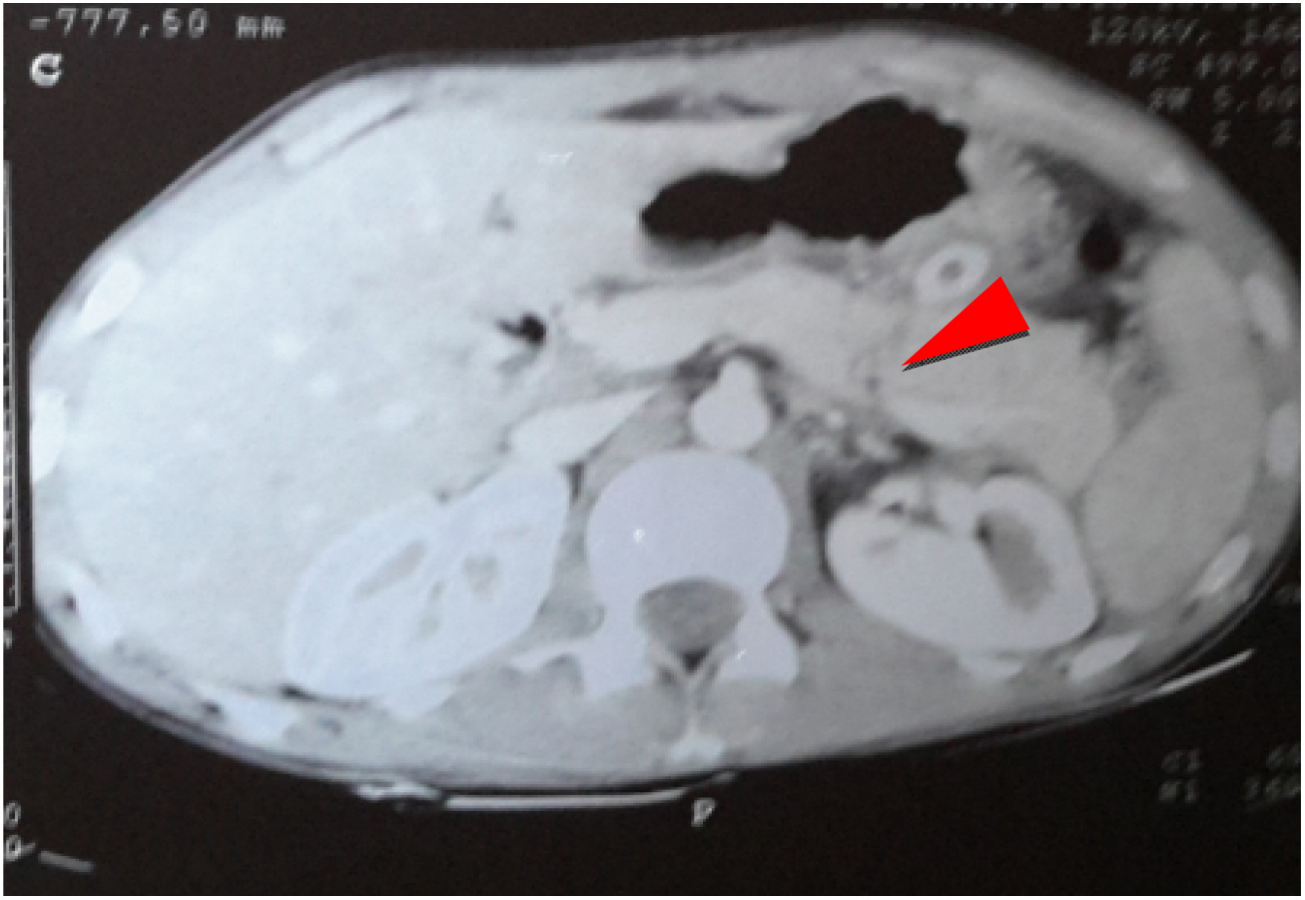
CT scan showing pancreatic body transection involving the duct (Red arrow), with distal pancreatic edema

Two weeks after admission, contrast abdominal CT scan was done, and showed transection of body of the pancreas involving the major duct with minimal pancreatic tissue loss and peripancreatic, perigastric, and perinephric fluid collection. Imaging assessment was Grade III Isolated pancreatic injury. Subsequent decision was made for delayed pancreatectomy and patient was continued on follow‐up at the ward (Figure 3).

Laparotomy was done on the 23rd postoperative day, and the intraoperative findings were transection of pancreatic body along with the major pancreatic duct and retroperitoneal patchy necrosis. With these findings, spleen preserving distal pancreatectomy was done and the duct was suture ligated and the capsule was over‐sewn. A suction drain was left in the lesser sac, the abdomen was closed, and the patient was transferred to back to the general wards. Patient had a stable postoperative course and was discharged on the 7th postoperative day with drainage in place. On the 14th postoperative day, the drainage tube was removed at the outpatient clinic after the output became nil, and he was discharged from the follow‐up clinic.

## 3 REVIEW OF LITERATURE

Blunt pancreatic injuries is a very rare entity occurring in 0.3% of the trauma population.^5^ Five percent of blunt abdominal trauma patients have pancreatic injuries.^6^ Motor vehicle accidents (steering wheel and seat‐belt impacts) in adults, and motor vehicle and bicycle handlebar injuries in pediatric age groups are the most common causes of blunt pancreatic injuries.^7^, ^8^, ^9^ Contact sport related pancreatic injuries have also been sporadically reported.^10^, ^11^ Domestic violence related to severe pancreatic injury secondary to a punch to the abdomen has also been reported in a single publication.^12^ To the best of our knowledge, this is the first case of isolated blunt injury following a homicidal kick injury to the abdomen.

Reports consistently showed that isolated pancreatic injuries from blunt trauma mechanisms delay to present with symptoms for at least 24–48 h.^13^, ^14^, ^15^ Delay in diagnosis of blunt pancreatic injury patients seems to increase with intoxication upon admission, low injury severity score, low abdominal trauma severity index, isolated pancreatic injuries, and initial non‐operative management decisions.^16^ This delay is associated with increase in morbidity and mortality.^17^ In our patient, the diagnosis was not suspected and investigation was not done at admission as the diagnosis was made intraoperatively.

Pancreatic ductal injuries are associated with higher pancreas specific morbidities, and mortality.^18^, ^19^ This fact serves to confirm that American Association for Surgery of Trauma (AAST) organ injury scale for pancreatic injuries is a valid method to stratify pancreatic injuries.^20^, ^21^

Controversies exist on whether pancreatic enzymatic markers like the serum amylase and Lipase, could serve as a screening modality for early detection of pancreatic injuries.^22^ In as many as 40% of the patients, initial level of serum amylase maybe normal. But would subsequently increase in up to 90% of blunt trauma patients presenting with a pancreatic injury.^23^ Similarly, for patients arriving from trauma scene with blunt mechanism, hyperamylasia was shown to be associated with increased mortality rate, traumatic brain injury, facial injury, hollow viscous injury, and pancreatic injury, but was non‐specific in accurately predicting pancreatic injury.^24^ Similarly, Moretz et al. failed to find an association between the serum amylase levels and pancreatic injuries in blunt trauma population.^25^ On the contrary, Mahajan et al. prospectively reported that at the cutoff time of 3 h from the time of injury, the sensitivity and specificity of combined use of serum amylase and lipase evaluation was 85% and 100%, respectively.^26^ If screening of blunt trauma with enzyme markers is to be done, it would be better to perform both markers together rather than only serum amylase. These tests were not done in our patient preoperatively since pancreatic injury was not suspected. The serum amylase and lipase done after the first laparotomy were significantly elevated.

Contrast‐enhanced CT scan of the abdomen had been the mainstay of imaging workup in blunt trauma patients with a stable hemodynamic status.^27^ Contrast‐enhanced CT scan in most studies have had a sensitivity and specificity of <80%, although this is likely to improve with the widespread use of multidetector CT and thinner collimations.^28^ MRI with MR pancreatography has recently been presented as a problem‐solving tool for diagnosis of pancreatic duct disruption.^27^, ^29^ Particularly for ductal disruption, the sensitivity of MR pancreatography is between 90% and 100%.^30^ In a low‐income institution, the value of abdominal ultrasound should be evaluated for diagnostic sensitivity and specificity for blunt pancreatic injuries. The largest report on diagnostic sonography for pancreatic injury done by Sato and Yoshi^31^ on 299 patients showed sensitivity of 44% and specificity of 100%. With such a low sensitivity and specificity, ultrasound cannot serve as a replacement for CT scan and MRI. Reports have been brought up on contrast‐enhanced ultrasonography and intraoperative ultrasonography for the diagnosis of pancreatic duct disruption but at present the place of these modalities is yet to be elucidated.^32^, ^33^

The treatment for pancreatic injuries has been evolving over the past decades.^34^ The tendency to operate on pancreatic trauma patients, especially in isolated pancreatic injuries has been decreasing since the 1990s.^35^ Non‐operative management is now practice of choice in most trauma centers for low grade, AAST organ injury score (OIS) I and II, patients.^35^, ^36^ On the contrary, for AAST OIS grade III and IV injuries, Koganti et al.^37^ showed only 29.4% were successfully managed non‐operatively. Higher failure in non‐operative management was seen in patients with multiorgan injuries, ileus, necrotizing pancreatitis, and contusion on CT scan.^37^ Similarly, Beres et al.^38^ reported, in patients with grade III and higher pancreatic injuries, non‐operative management was associated with increased complications rate, length of hospital stay and time on total parenteral nutrition by 13 days. In addition, the Eastern Association for the Surgery of Trauma (EAST) group analyzed 4 of the large comparative studies with operative and non‐operative approach to pancreatic trauma. The conclusion was that even though the individual studies did not have complications rate between operative and non‐operative groups differ in statistically significant manner, the grouped outcomes of all the studies may have. For these reasons, the practice guideline recommended operative intervention for AAST OIS grade III and IV pancreatic injury patients.^39^

Distal pancreatectomy is the procedure of choice for patients with pancreatic trauma at or distal to the superior mesentric vessels with acceptable mortality and morbidity rates.^40^ Debate on whether the spleen should be preserved or not has been going on for long, especially in patients with isolated pancreatic injuries, Lin et al. compared spleen preservation versus splenectomy and were able to show higher rate of re‐intervention rate for complications in Splenectomy group.^41^ This is an observational study with potential for high bias with a notable subgroup variability, so a conclusive recommendation is difficult to make. Nonetheless, the rule of thumb should be preserving the spleen if technical and physiological conditions are permitting. In our patient, spleen preserving distal pancreatectomy was performed uneventfully.

Another major area of confusion would be management of hemodynamically unstable blunt abdominal trauma patients with pancreatic injuries undergoing damage control laparotomy. Studies have shown that AAST OIS grade III injuries could undergo distal pancreatectomy at the index damage control surgery or in a delayed fashion after packing and drainage had hemodynamically stabilized the patient.^42^, ^43^, ^44^ The level of evidence is quite poor to advice for or against primary distal pancreatectomy at this time. But, near complete body or tail transections of the pancreas, or major ductal injury of the body of the pancreas can undergo distal pancreatectomy provided that bleeding control is successful and experienced surgeon is available. If decision is made for delayed intervention, packing with suction drainage is a better option. Pancreatic ductal ligation only approach in a damage control setting is not supported by evidence.

With regards to the outcome, severe pancreatic injury patients have a high morbidity and mortality. The largest registry‐based study of pancreatic trauma from US reported a 21.2% overall mortality rate, and 17.8% among the blunt trauma subgroup. Similarly, the morbidity rate was reported to be as high as 53%.^45^ Pneumonia, sepsis, and wound infections are the most common complications in group of patients with blunt pancreatic trauma.^5^ Fortunately, our patient did not develop any of these complications.

Finally, pitfalls in the management of the patient presented needs a brief discussion. In our opinion, initial abdominal contrast CT scan at initial admission would have aided with early diagnosis. Additionally, during initial laparotomy, which was performed 48 h after admission, distal pancreatectomy could have been attempted as there was no evidence for hemodynamic instability, mandating a damage control route. Based on the available evidence, the interval duration of time between the first and definitive surgeries was prolonged, and patient could have benefited from earlier re‐laparotomy and earlier discharge, although the argument for delay was possible spontaneous closure of the pancreatic fistula.

## 4 CONCLUSION

The possibility of isolated pancreatic injury should be considered in the differential diagnosis of intraabdominal solid organ injuries even in patents with “trivial” blunt abdominal trauma. If clinical evaluation and investigations are not revealing as to the organ involved in the injury, further investigation directed to the pancreas should be considered, especially in conditions with worsening of the clinical condition. Serum amylase, lipase, abdominal CT, and MRI are possible options in the further workup. For patients with grade III–V injuries, the preferred approach is to do surgery primarily, and specific to grade III injuries, the preferred approach should be distal pancreatectomy. Choice in spleen preservation is left to the discretion of the surgeon to be decided based on technical and physiologic parameters. In damage control situations, distal pancreatectomy can be done in select cases. In situations where delayed definitive care is planned, packing with suction drainage is a preferred approach.

## AUTHORS CONTRIBUTIONS

S.K wrote the case presentation and literature review. G.T gathered pictures for the publications, wrote the initial draft of the case presentation and edited the manuscript.

## ETHICAL APPROVAL

This manuscript was written according to the world medical association declaration of Helsinki.

## CONSENT

Consent for publication was acquired from the patient.

## Data Availability

Data for this case report would be available upon reasonable request to the authors.
